# Diverse types of knowledge on a plate: a multi-perspective and multi-method approach for the transformation of urban food systems towards sustainable diets

**DOI:** 10.1007/s11625-022-01287-9

**Published:** 2023-02-10

**Authors:** Marta López Cifuentes, Marianne Penker, Lisa Kaufmann, Fritz Wittmann, Valentin Fiala, Christina Gugerell, Christian Lauk, Fridolin Krausmann, Michael Eder, Bernhard Freyer

**Affiliations:** 1 Department of Economics and Social Sciences, Institute for Sustainable Economic Development, University of Natural Resources and Life Sciences (BOKU), Feistmantelstraße 4, 1180 Vienna, Austria; 2 Centre for Environment and Sustainability, University of Surrey, Arthur C Clarke Building, Guildford, GU2 7XH UK; 3 Department of Economics and Social Sciences, Institute of Social Ecology, University of Natural Resources and Life Sciences (BOKU), Schottenfeldgasse 29, 1070 Vienna, Austria; 4 Department of Economics and Social Sciences, Institute of Agricultural and Forestry Economics, University of Natural Resources and Life Sciences (BOKU), Feistmantelstraße 4, 1180 Vienna, Austria; 5 Research Center for Sustainability, Freie Universität Berlin, Ihnestraße 22, 14195 Berlin, Germany; 6 Division of Organic Farming, Department of Sustainable Agricultural Systems, University of Natural Resources and Life Sciences (BOKU), Gregor-Mendelstraße 33, 1180 Vienna, Austria

**Keywords:** Scenario approach, Transdisciplinary research, Interdisciplinary research, Urban food systems, Sustainable diets, Knowledge co-creation

## Abstract

Urbanization processes are accompanied by growing global challenges for food systems. Urban actors are increasingly striving to address these challenges through a focus on sustainable diets. However, transforming food systems towards more sustainable diets is challenging and it is unclear what the local scope of action might be. Co-production of knowledge between science and non-science is particularly useful for analysing context-specific solutions and promise to result in more robust socio-economic, political and technical solutions. Thus, this paper aims to integrate different types and sources of knowledge to understand urban food systems transformation towards a more sustainable diet in Vienna; and, second, to analyse and reflect on the difficulties and ways forward to integrate diverse actors’ perspectives, multiple methods and epistemologies. We created different future scenarios that illustrate the synergies and trade-offs of various bundles of measures and the interactions among single dimensions of sustainable diets. These scenarios show that there is plenty of scope for local action, but co-ordination across diverse groups, interests, and types of knowledge is necessary to overcome lock-ins.

## Introduction

Urban food systems are becoming places for experimentation, where urban dwellers, policymakers and businesses are developing novel ways to support a transformation towards more sustainable production and consumption patterns. The UN Agenda 2030^1^ calls for a bold transformation to move the world onto a sustainable path. For that, it is needed to counteract the increasing global challenges that the dominating industrialized food system is triggering and facing—from climate change and resource scarcity to social inequalities (see, for example, Rockström et al. 2009; Tilman and Clark 2014; Campbell et al. 2017). In this regard, sustainable diets are often seen as a key factor to enhance urban food systems’ long-term capacity for food and nutrition security while improving the health of people and the planet (Garnett 2011; Johnston et al. 2014; James and Friel 2015).

There are different interpretations of sustainable diets depending on the local context (Clonan and Holdsworth 2012), leading to a broad range of strategies that are discussed to make diets and corresponding food systems more sustainable. Here, we focus on three prominent dimensions of sustainable diets (e.g. Bere and Brug 2009; Lacour et al. 2018): reduced meat consumption, increased consumption of organic food products and increased consumption of regional food. These dimensions, however, contribute differently to sustainable food systems and trade-offs between their ecological, social and economic impacts have to be considered (see, for example, Born and Purcell 2006; Garnett 2011; Kopainsky et al. 2020; Helander et al. 2021).

The study of sustainable diets has many facets and wide-ranging ramifications within research. Most studies focus on only one aspect of sustainable diets such as people’s motivations to change dietary patterns (e.g. Zur and Klöckner 2014), the economic impacts of a conversion to organic agriculture (e.g. Kerselaers et al. 2007) or the environmental footprints of individual food items (e.g. Geibel et al. 2021). However, the local scope of action towards more sustainable urban food systems is a complex societal challenge that demands a rethinking of research approaches.

Transdisciplinary approaches have emerged as key components of sustainability studies. As argued by Brandt et al. (2013), sustainability studies need to understand unprecedented and interconnected challenges and, thus, require cooperation between different scientific domains and society at large. Although there are different definitions and interpretations of its meaning (Pohl et al. 2021), transdisciplinarity is generally characterized by the integration of various scientific disciplines that focus on shared problems and of non-academic practitioners (for a review, see, for example, Brandt et al. 2013; Lawrence et al. 2022). Going beyond a simple exchange of views, such transdisciplinary collaborations emphasize active research cooperation among diverse actors and the co-creation of knowledge (Lawrence et al. 2022).

Actors from the broader society are aware of socio-political issues and have specific local knowledge and experience that scientists often lack. Thus, the co-production of knowledge between science and non-science is particularly useful for analysing context-specific solutions to complex socially relevant problems (e.g. environmental sustainability) and promises to result in more robust socio-economic, political and technical solutions that are socially accepted and better adapted to the particular context (Pohl et al. 2010; Raymond et al. 2010; Enengel et al. 2012).

In contrast to disciplinary research, the transdisciplinary co-creation of an understanding of a problem and promising ways of dealing with it means involving actors with diverse epistemologies in the process (Bammer 2019). Despite the growing literature on empirical experiences uncovering the possibilities and challenges of transdisciplinary approaches in sustainability studies (for example, Slater and Robinson 2020; Scholz and Steiner 2015), so far, there is no broadly accepted framework for analysing and comparing knowledge co-creation (Enengel et al. 2012; Mauser et al. 2013; Scholz and Steiner 2015; Muhar and Penker 2018; Pohl et al. 2021) This lack of conceptualisation hampers the further development of transdisciplinary research and the knowledge exchange between disciplines that do not share methodological or conceptual definitions (Brandt et al. 2013; Bammer 2019).

To contribute to the literature on knowledge co-creation and, more broadly, transdisciplinary research, this paper has two aims: first, to integrate different types and sources of knowledge to understand urban food systems transformation towards a more sustainable diet in Vienna; and, second, to analyse and reflect on the difficulties and ways forward to integrate diverse actors’ perspectives, multiple methods and epistemologies.

In a first step, the inclusion of different types of knowledge in this transdisciplinary research allowed us to understand the local scope of action for a transition towards a more sustainable diet in Vienna. We integrated multi-disciplinary scientific and non-academic knowledge into an inter- and transdisciplinary research framework. This approach facilitated an extended knowledge production process that included a manifold of actors and different forms of information produced by the ‘scientific’ and ‘lay’ communities (Mobjörk 2010). This approach aimed to answer the question: ‘What are possible pathways for future transitions towards a more sustainable diet in an urban context?’ To this end, we integrated a wide range of results from different disciplines and the perspectives of different local actors concerning the three dimensions of sustainable diets investigated in this project and use them to discuss three different future scenarios and the pathways to reach them.

The second step was to reflect on the experiences from this research process by addressing the question ‘who can contribute what kind of knowledge in which phase of a transdisciplinary project and why?’ by using Muhar and Penker’s (2018) framework of knowledge co-production. This framework was chosen due to its suitability for the ex-post analysis of knowledge co-production in transdisciplinary research processes.

In this paper, we first present the inter- and transdisciplinary approach and methods used in the project. Bio-physical (ecological) and farm-economic modelling served as the core of the research design. This approach is embedded into a socio-scientific-transdisciplinary bracket with a system and scenario approach that specifically draws on the knowledge of the local food actors, identified and reflected via interviews, surveys and workshops. We then introduce the analytical framework to reflect on the experiences of the research process. After that, we present the results and different types of knowledge generated. Finally, we integrate the generated knowledge in the form of future scenarios, followed by a critical reflection on knowledge co-production and a conclusion.

## Mixed methods: an inter- and transdisciplinary approach

This section presents the methods applied within the research project ‘the future of urban food’^2^ that were used to answer the first research question (i.e. *what are possible pathways for future transitions towards a more sustainable diet in an urban context?*).

### Organizational setting: ‘The future of urban food’ project

This paper is the outcome of the final reflections on the experiences and results from the project ‘The future of urban food’. The project started in 2018 and lasted 4 years. It aimed to investigate the impact of changes in urban food systems and urban food consumption patterns on agriculture and the environment and thereby start a social discourse on the future development of urban food systems. To this end, we focused on the city of Vienna, the capital of Austria, which is a fast-growing city with currently almost two million inhabitants. We used this city as a case study for a major European city with a broad range of food initiatives and a diverse agricultural hinterland. Furthermore, the City of Vienna aims to enhance sustainable urban food policies—Vienna is part of the Milan Urban Food Policy Pact^3^ and the European Organic Cities Network.^4^ While in the project proposal, we aimed at scoping the potential of a Viennese Food Policy Council,^5^ such a civil society association with members from production up to consumption was already founded in the meantime. This non-governmental organization aims to make Vienna’s urban food system more sustainable and democratic.

Four research institutes representing different disciplines were involved in this project. Two institutes mainly focused on the bio-physical and farm-economic modelling part of the project, while the other two focused on the socio-political aspects (Table 1).

**Table 1:** Project steps, methods and outcomes

Project phase	Project steps	Methods	Outcomes	Timeline
Food system modelling	Bio-physical modelling and analysis	Spreadsheet-based accounting model that translates food intake into primary biomass and land use on different spatial scales and calculates emissions for the different processes along the value chain	Land and greenhouse gas (GHG) footprint of Vienna’s UFS	March 2018
			Quantification of effects of changes in consumption on footprints.	
	Farm-economic modelling and analysis	Farm model using farm types of the surrounding areas of Vienna that operate with linear programming and adaptive responses from a survey of farmers to characterize economic and environmental impacts on farms	Land-use change of the region 100 km around Vienna within Austria	
			Quantification of gross margins, workload, plant nutrient supply of farms	
Transdisciplinary food system analysis	System analysis	Qualitative interviews (regime: *n* = 35; niche: *n* = 21), three focus groups (*n* total = 17), participatory workshop (*n* = 48), online survey with Vienna’s UFS actors (*n* = 23), and surveys with community-supported agriculture members in Austria (*n* = 78), Norway (*n* = 88) and Japan (*n* = 43)	Structural and functional analysis of Vienna’s UFS	
			Identification and definition of drivers of change and their relevance in Vienna’s UFS	
	Actors' attitudes towards sustainable diets	Three online surveys with Viennese population, foci: regional food (SR, *n* = 378); organic food (SO, *n* = 377); meat (SM, *n* = 374)	Report on the perceptions of different actors of Vienna’s UFS (incl. population and farmers) on SDs	
		Online survey with farmers (100 km around Vienna within Austria) (*n* = 1720)		
		Qualitative interviews with Vienna’s UFS actors (*n* = 38)		
		Focus group with Caritas community cooking (*n* = 6)		
Transdisciplinary scenario process	Scenarios development	Role game with bachelor students from BOKU university (*n* = 20)	Test scenarios	December 2019
		Online workshop with research team (*n* = 12)	Three future scenarios for Vienna’s UFS in 2040	
	Consistency and robustness check	Online survey with research team (*n* = 12)	Reflections on consistency and robustness regarding future threats and opportunities	October 2020
		Online workshop with research team, advisory board and the Viennese food policy council (*n* = 19)		
	Transition pathways	Online workshop with research team (*n* = 12)	Recommendations for action	November 2020

The project was also designed as a transdisciplinary project through the involvement of an advisory board (Table 2). The advisory board consisted of a group of 14 representatives from the private food sector, city administration, interest groups and civil society (including representatives from the food policy council). Board members were involved in essential project steps through a consultation process and participated in the knowledge co-creation for the system analysis and scenario development (Table 2). The knowledge from various actors of the Viennese urban food system helped to embed the quantified results of the models into the local and national structural context and by doing so, hopefully, increase their value for the urban food system transformation of Vienna and its surroundings.

**Table 2:** Involvement and role of different stakeholders in the project

Project steps	Type of involvement	Stakeholders	Role of stakeholders in the project
Planning and definition of goals	Project meeting 05/2018	Advisory board	To co-develop the guidelines for the joint cooperation during the project (including motivations, expectations and organisational issues)
Bio-physical modelling and analysis	Project meeting 05/2018	Advisory board	To assess the model and provide missing data on the spatial location of value chains for certain product groups relevant for the project
	Project meeting 09/2019	Advisory board	To give feedback on preliminary results and their consistency
Farm economic modelling and analysis	Project meeting 11/2018	Advisory board	To provide context-specific knowledge to increase the survey’s comprehensibility and thus the response rate
	Project meeting 09/2019	Advisory board	To give feedback on preliminary results and their consistency
System analysis	Project meeting 05/2018	Advisory board	To provide expert knowledge on a first draft of Vienna’s UFS model and identify relevant context-specific actors To provide context-specific knowledge on innovative food initiatives and co-select examples for further analysis
	Interviews 2018–2019 and online survey	Advisory board and Vienna’s UFS actors	To provide further expert knowledge on Vienna’s UFS and for the implementation of sustainable diets in Vienna
	Project meeting 11/2018	Advisory board	To identify context-specific opportunities and challenges in the cooperation between established actors and new food initiatives
	Open workshop 01/2019	Vienna’s food policy council, Vienna’s UFS actors and interested citizens	To discuss final results on Vienna’s drivers of change for the implementation of sustainable diets and co-define missing ones
Actors' attitudes towards sustainable diets	Interviews 2018–2019	Advisory board and Vienna’s UFS actors	To share context-specific and expert perspectives on sustainable diets and their implementation in Vienna’s UFS
	Surveys 2019	Farmers and citizens	To provide personal perspectives on sustainable diets
Scenarios development	Project meeting 05/2019	Advisory board and Vienna’s food policy council	To work on first visions for Vienna’s UFS and to give feedback on the planning of the scenario workshop
Consistency and robustness check	Project meeting 11/2020	Advisory board	To provide feedback on the consistency of the developed scenarios and to participate in their robustness check
	Online assessment 2020	Advisory board	To co-assess the consistency of the developed scenarios and to check their robustness
Transition pathways	Workshop 11/2020	Vienna’s food policy council	To discuss possible measures for the transition pathways of the three scenarios and their food strategy
	Workshop 11/2021	Community kitchen (Caritas)	To discuss possible measures for the transition pathways of the three scenarios
Final results and reflections	Workshop 12/2021	Advisory board	After presentation of final results from the scientific team: To discuss final results of the project and identify inconsistencies To co-reflect on the collaboration throughout the project and meaningfulness of the project

*UFS* urban food system

To assess the effects of different food intake, we used three dietary patterns characterized by a certain share of meat and dairy products: the diet as usual (i.e. Austrian average diet in 2015) with 65 kg/year of meat and 110 kg/year of dairy products (Statistik Austria 2020, 2021); the EAT-Lancet planetary health diet with 15 kg/year of meat and 88 kg/year of dairy (Willett et al. 2019); and the recommended diet by the Austrian Nutrition Society with 22 kg/year of meat and 195 kg/year of dairy (BMG 2015; Rust et al. 2017).

### Food system modelling

We used bio-physical and farm-economic models to quantify and assess the impacts of changes in the three dimensions of sustainable diets on Vienna’s urban food system (e.g. on resource flows and farming systems) (Table 1). The bio-physical model applied a systems-based approach that translates food intake into primary biomass and land use on different spatial scales and calculates GHG emissions for the different processes involved along the value chain (in particular agricultural production, transport, food processing) (for further details see Lauk et al. 2022). In a counterfactual approach, we used the model to explore how changes along the three dimensions of sustainable diets impact the land and GHG footprint of urban food consumption. Further, the farm-economic model based on linear optimization and switches between farms of different farm types (i.e. sets of individual farms that are relatively homogenous in size, intensity, land use and specialization) was used to simulate changes in the agricultural production systems and their impact on product output and gross margins of farms in the regional hinterland of Vienna (see Wittmann and Eder, Forthcoming). A major challenge of quantitative modelling of the Viennese food system was a surprising lack of systematic data on prevailing food consumption patterns and urban supply chains (see Lauk et al. 2022).

### Transdisciplinary food system analysis

A system analysis from a multi-actor, multi-level perspective^6^ (Geels 2002) involved professional practice experts and strategic case actors from the regime level (i.e. representatives of incumbent food organizations on the local and national levels) and local case actors from niche organizations (i.e. representatives of new organizations experimenting with sustainability innovations) (Table 1). This approach allowed us to map Vienna’s urban food system and gain a holistic understanding of the actors and driving forces of the system. Qualitative interviews and workshops were used to explore the perspectives of professional practice experts in ministries, NGOs, and businesses active beyond the local level, as well as strategic case actors—i.e. representatives from the city administration, the Viennese food policy council and local case actors such as food businesses (see López Cifuentes et al. 2021). To include the perspectives of disadvantaged groups regarding Vienna’s urban food system and sustainable diets, a focus group was also organized with members of the Caritas^7^ community cooking project.

We further surveyed the attitude towards the defined dimensions of sustainable diets among the Viennese population and farmers in and around the city (100 km radius of Vienna within Austria) (Table 1). Quantitative data from the farmers’ survey was analysed using a binary logit model—i.e. a form of a logistic regression analysis that estimates the probability of an event occurring with a dichotomous dependent variable (Cramer 2003)—and several steps were followed to model farm type adaptation (see Wittmann and Eder Forthcoming). For the Viennese population, three surveys were conducted (one per sustainable diet dimension to keep the number of questions manageable for respondents and increase the response rate). A seven-point Likert scale that offered seven different options to choose from for each statement to be assessed (Field 2009) was used. Then, survey data were analysed using descriptive and multi-linear regression (MLR)^8^ analyses. Furthermore, surveys of niche actors in six cities in Norway, Japan and Austria, helped to differentiate between context-specific results and those that hold across urban contexts (see Gugerell et al. 2021). Qualitative data from workshops, interviews and open questions from surveys were analysed using inductive and deductive coding (Saldana 2009).

### Transdisciplinary scenario process

**Fig. 1:**
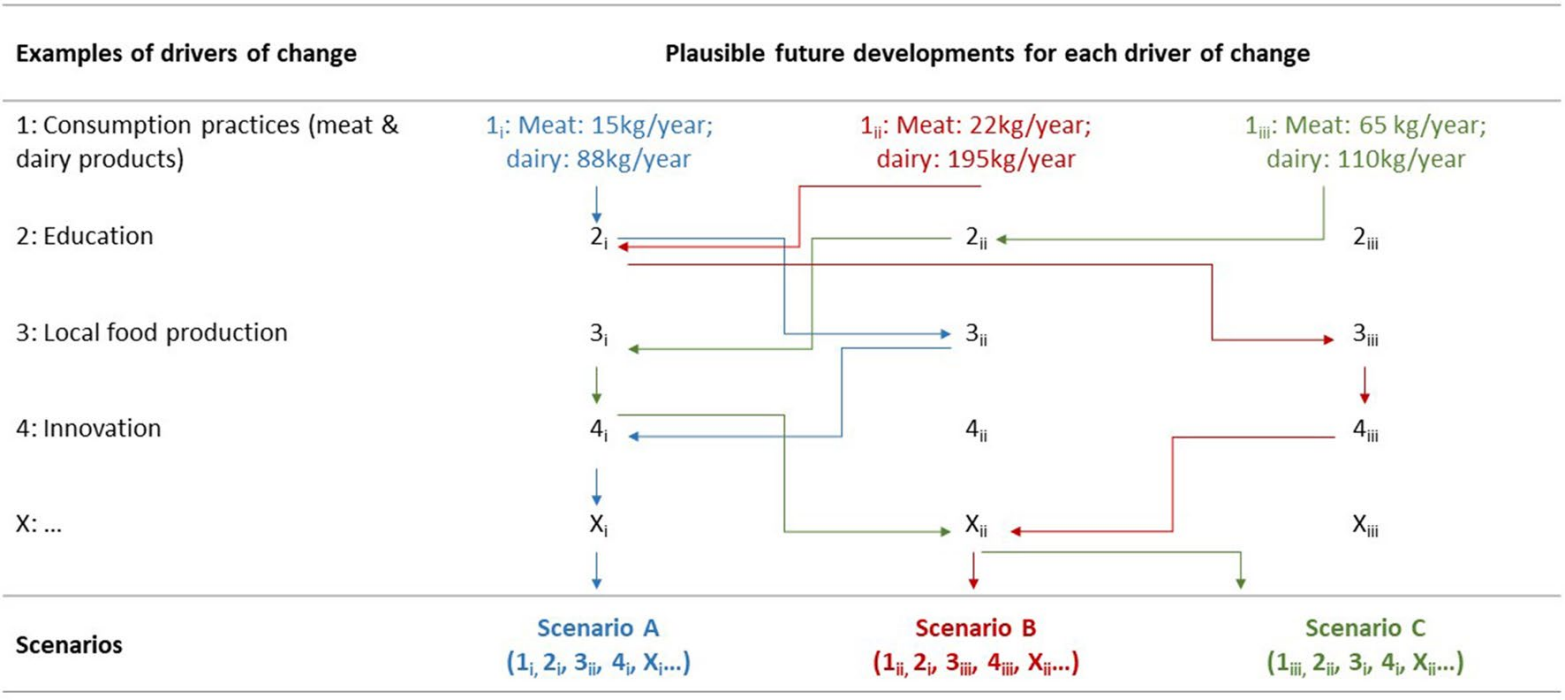
Linking different drivers developments (options i, ii, iii) to plausible and coherent scenarios for Vienna's urban food system. Arrows: each colour represents a starting point for Driver 1 and combines the other drivers into one scenario. Source: adapted from Penker and Wytrzens (2011, p. 181)

The integration of diverse actors’ knowledge is one of the core functions of scenario processes (Börjeson et al. 2006; Wiek et al. 2006) and their assessment of possible courses of action makes participatory scenario processes a suitable instrument for inter- and transdisciplinary research. Furthermore, scenario processes can facilitate targeted intervention in future developments and serve as a spatial-strategic planning tool (Penker and Wytrzens 2011; Schauppenlehner-Kloyber et al. 2013). In this project, the goal was to study how Vienna’s urban food system might look in the year 2040. The scenario process helped to integrate the empirical knowledge from qualitative and quantitative research, context-specific and phenomenological knowledge from strategic and local case actors, generalizable knowledge from professional practice experts, literature, or cross-country comparisons, as well as the actor’s strategic knowledge on key-actors and the transformability of food systems. Based on trends analysed through secondary data and literature, we assessed plausible options of how key drivers of Vienna’s urban food system identified in the system analysis (see above) could change until 2040 (Fig. 1), which is the year the Austrian government targeted for carbon neutrality. The possible future developments of the first driver of change, i.e. consumption practices concerning meat and dairy products, were predefined by the scientists based on a technology-optimistic outlook that could allow keeping the status quo diet with 65 kg of meat and 110 kg of dairy per person and year (Statistik Austria 2020, 2021), the recommendations by the Austrian Ministry of Health for a healthy diet with 22 kg meat and 195 kg of dairy (BMG 2015; Rust et al. 2017) and the planetary health diet with 15 kg meat and 88 kg dairy (Willett et al. 2019). For the other drivers of change, three plausible future developments were suggested by participants of the scenario workshop. Then, based on a consensus-oriented discussion, three consistent scenarios were developed by combining plausible combinations of different developments of the drivers of change as shown in Fig. 1.

Based on this dialogue-based method, we developed scenarios of three alternative futures that are as consistent, plausible and as divergent as possible (for a more detailed description of the method, see e.g. Gausemeier et al. 1998). After the workshop, scientists and two food council members contributed individual consistency checks [see Seeve and Vilkkumaa (2022) for a description] to improve the consistency of the scenarios. Furthermore, the members of the advisory and the food policy council assessed the relevance of certain threats (e.g. extreme climate events) and opportunities (e.g. preservation of regional agro-ecosystems) for the different scenarios to distinguish between scenarios that are considered more or less robust. Finally, possible measures for transition pathways were co-produced with members of the food policy council for each scenario based on previously collected data—i.e. interview transcripts, project meetings' and workshops’ protocols and discussions with other scientists.

The method adapted for the Viennese context was first tested with a group of students and then applied in a participatory process with the scientific team, the city administration and the Viennese food policy council. The newly founded Viennese food policy council and their collaboration with the City of Vienna opened a window of opportunity for linking the scenario process for transdisciplinary knowledge co-production with the development of a food strategy, which was also intended by the two actor groups. And indeed, in a series of workshops with the food policy council and city representatives from the department of environmental protection, we planned a participatory scenario process with the broad involvement of a manifold of actors to inform the food strategy. Co-funding was confirmed, rooms were booked and invitation letters were sent out when the city decided to cancel the participatory scenario process with the official justification of ‘organizational reasons’. Therefore, the final scenario process focused on the scientists of the project, the project’s advisory board and the Viennese food policy council. In the end, the outcomes informed the food strategy via the participation of the city administration and food council representatives in this process. As the Vienna food strategy is intended to motivate farmers, food businesses, civil society and city administration to contribute to an urban food sustainability transition, we put a focus on scenarios that many Viennese food actors might consider reasonably attractive (in contrast to a worst case or business as usual scenarios).

## Analytical framework for an ex-post analysis of knowledge co-production

To reflect on the experiences from a transdisciplinary project and answer our second research question (i.e. *who can contribute what kind of knowledge in which phase of a transdisciplinary project and why?*), we refer to the framework of Muhar and Penker (2018), which originates from Enengel et al. (2012). In a small but growing family of frameworks for the evaluation of transdisciplinary research, it provides a heuristic to analyse the knowledge integrated by diverse actor groups (Muhar and Penker 2018). Its actor focus distinguishes it, for example, from the transdisciplinarity wheel that visualizes and discusses three elements of transdisciplinary research—context, process and product—and implications for research design, execution and quality evaluation (Carew and Wickson 2010; Wolf et al. 2013; Scholz and Steiner 2015; Luederitz et al. 2017). Other frameworks provide support in evaluating sustainability transition experiments (Luederitz et al. 2017) or societal impact (Wolf et al. 2013). Some of them focus more on an ex-ante support of the research design (for example, Mauser et al. 2013; Radinger-Peer et al. 2022), while we looked for an actor-focused ex-post evaluation of knowledge co-production. The chosen framework consists of four elements:

- Who: the framework differentiates between: *core scientists* of the project team; *scientific consultants* that provide scientific support from outside the project team; *professional practice experts* outside Vienna’s urban food system who are familiar with the practical and political aspects of the issue at hand (in this case experts in political parties, NGOs and research institutions with specific expertise on sustainable diets and food systems); *strategic case actors* who hold formal or informal responsibilities in Vienna’s urban food system (businesses, interest groups, municipal departments); and other *local case actors* that are either personally affected by or involved in, the local case of Vienna.
- When: the research project steps in which knowledge is (co-)produced (see Table 1 in the next section).
- What: The framework describes knowledge contributions based on the following three dimensions: (1) scale dimension (*context-specific knowledge* refers to the concrete setting of the Viennese case and *generalized knowledge* claims to be universally valid and is expressed in a systematic way, free from context-specific conditions and constraints); (2) functional dimension (*phenomenological knowledge* addresses (local) social and environmental phenomena and *strategic knowledge* focuses on connections and interrelations of system elements); and (3) epistemological dimension (*experiential knowledge* is derived from one’s own life experience or traditional knowledge and is often tacit or implicit and *scientific knowledge* is based on empirical evidence or scientifically acknowledged theories).
- Why: the goals of involving diverse groups can vary for the actor groups and in the different research phases and they are also closely linked to the method, such as information (presentation of research objectives on an advisory board), consultation (feedback by advisory board and other actors) and co-decision-making (e.g. robustness check in a workshop setting).

This framework was used for the ex-post analysis of the experiences from our transdisciplinary project (see Sect. 2.1). First, the actors that were involved in the project were classified depending on their role according to the first element of the framework (‘who’). Second, we identified the different types of knowledge created and integrated during the project from scientific analysis (i.e. interview transcripts, surveys and modelling results) and consulting processes (i.e. protocols from meetings and workshops) and deductively analysed them according to the knowledge categories and actor groups defined in the framework. Finally, we reflected how this knowledge was integrated into the transdisciplinary process by using minutes of project meetings involving stakeholders and e-mail exchanges.

## Involved actor groups, forms of integration and types of knowledge

In this section, we present the results from the analysis for how actors were involved in the different steps of the project (Table 3).

**Table 3:** Type and number (*n*) of actors involved in each research phase

Project steps	Core scientists (involved disciplines)	Scientific consultants (involved disciplines outside project team)	Professional practice experts (outside the case)	Strategic case actors in the Viennese urban food system	Local case actors
Proposal writing	Social ecology (*n* = 2); agricultural economics (*n* = 1); organic farming systems (*n* = 3); rural sociology (*n* = 1)				
Bio-physical modelling and analysis	Social ecology (*n* = 4)	Agricultural economics (*n* = 3); livestock sciences (*n* = 1)	National statistical office (*n* = 1); research institution (*n* = 1); interest group (*n* = 1)		Food business (*n* = 1)
Farm-economic modelling and analysis	Agricultural economics (*n* = 2)	Agricultural science (*n* = 6)	Farmers (*n* = 7); agricultural chambers (*n* = 2)	Federal Ministry of Agriculture, Regions and Tourism (*n* = 1)	
System analysis	Organic farming systems (*n* = 3); rural sociology (*n* = 2)	Agricultural science (*n* = 3); food policy (*n* = 1)	National NGOs, research institutions, politicians and interest groups (*n* = 13)	City administration and Viennese food policy council (*n* = 9)	Farmers, food industry and food businesses (*n* = 82)
Actors’ attitudes towards sustainable diets	Organic farming systems (*n* = 3)	Social economy (*n* = 2)	National NGOs, research institutions and interest groups (*n* = 12)	City administration and Viennese food policy council (*n* = 9)	Viennese citizens, farmers, food industry and food businesses (*n* = 2862)
Scenario method test and scenario development	Social ecology (*n* = 3); agricultural economics (*n* = 2); organic farming systems (*n* = 3); rural sociology (*n* = 2)				Students (*n* = 20)
Consistency and robustness check	Social ecology (*n* = 3); agricultural economics (*n* = 2); organic farming systems (*n* = 3); rural sociology (*n* = 2)		National NGOs, research institutions and interest groups (*n* = 5)	City administration and Viennese food policy council (*n* = 2)	Food industry (*n* = 2)
Transition pathways	Social ecology (*n* = 3); agricultural economics (*n* = 2); organic farming systems (*n* = 3); rural sociology (*n* = 2)		National NGOs, research institutions, politicians and interest groups (*n* = 13)	City administration and Viennese food policy council (*n* = 9)	Farmers, food industry, and food businesses (*n* = 82)

Core scientists were involved in all research phases (Table 3). The problem identification and a preliminary research design and methodology were defined and developed by a group of core scientists during the proposal writing, with limited exchange with other scientists and actors. Single strategic case actors already expressed their willingness to collaborate in the phase of project proposal writing (e.g. the city administration’s representative coordinating the local government’s food activities and responsible for the implementation of the Milan Urban Food Policy Pact and some representatives of businesses and the local chamber of agriculture), who later became part of the advisory board. However, these actors were involved neither in framing the problem nor writing the proposal. Other actors were invited after the funding decision based on a consultation with the city administration’s food representative. Thus, with some exceptions, the advisory board was not involved in problem framing and project development but was informed about the project’s objectives and was constituted at the beginning of the project. They then took a consultative function in the following project phases. Members of the advisory board are included in the classification in Table 3, as well as other actors that were involved in the project, such as other academics, farmers and citizens.

Members of the advisory board reflected on the research design and methods by giving feedback on the models, identifying and ranking cases and interviewees and testing surveys. In interviews and workshops, they provided generalized knowledge of the wider problem field (professional practice experts and strategic case actors) as well as context-specific insights into local cases (strategic case actors and local case actors). For the overall research process and particularly for the system analysis and scenario analysis, they contributed valuable strategic knowledge (e.g. key drivers for and barriers to food system transformation, the transferability of experiences from abroad, measures promising leverage for change and potential for local implementation). They contributed to the interpretation of the results, the contextualization and robustness of the scenarios and the identification and formulation of context-specific recommendations. They also facilitated the communication with further professional practice experts as well as strategic and local case actors (outside the advisory board), e.g. by naming potential interview partners or participants of focus groups, workshops, interviews and surveys. While the advisory board members were involved over the full project duration, these actors were only consulted at specific stages of the project and contributed mostly context-specific, phenomenological and experiential knowledge.

## Multi-disciplinary knowledge generated

In this section, we briefly discuss the multi-disciplinary knowledge that resulted from the project shown in Table 4.

**Table 4:** Summary of multi-disciplinary results of each defined dimension of sustainable diets

	Regionalization of the UFS	Increase organic food share	Reduced meat consumption
*Bio-physical and farm-economic model analysis*			
Land demand	Food sourced as far as possible from regional production (100 km radius): reduction by 21%	100% organic food: increase by 57%	Austrian recommended diet: reduction by 21% Planetary Health Diet: reduction by 35%
GHG emissions	Food sourced as far as possible from regional production (100 km radius): reduction by 12%	100% organic food: reduction by 18%	Austrian recommended diet: reduction by 9% Planetary Health Diet: reduction by 33%
Economic impact	Total gross margin increases by 8% (under assumption of unchanging prices) Farm production patterns stay mainly unchanged	Total gross margin increases by 22% (under assumption of unchanging prices) and labour requirements by 14% Share of organic utilized area would increase from 16 to 52% Public payments disbursed would increase by 22%	Total gross margin increases by 7% (under the assumption of unchanging prices) The quantity of meat produced declines by 26% Public payments disbursed would increase by 7%
*Attitudes towards sustainable diets*			
Viennese population’s perspectives	80% of respondents think the consumption of regional food is good for the environment 29.4% of respondents reporting to consume less than 85% regional food have a (very) high intention to consume mainly regional food The availability of regional food and visiting farmers’ markets positively interrelate with the intention to mainly consume regional food	58.7% of respondents agree that consuming mainly organic food is good for the environment 22.7% of respondents reporting to consume less than 85% organic food have a (very) high intention to consume mostly organic food Trust in labelling positively interrelate with the intention of consuming mainly organic food	52.6% of respondents consider that consuming less than 400 g meat/week is good for the environment; 16.6% disagree 12.8% of respondents reporting to consume more than 400 g meat/week have (very) high intention to consume less meat—41.6% (very) low Cultural values and gender positively related with the intention of consuming less meat
Farmers’ perspectives	72% of respondents judge that they will be affected by the effects of a scenario with higher organic and regional consumption 66% of respondents would change their production orientation if regional consumption were higher	57% of respondents would change their production orientation if organic consumption were higher In the scenario with increased organic and regional food consumption, 44% of conventional farmers would convert to organic	45% of respondents judge that they will be affected by the effects of a scenario with higher regional food and lower meat consumption 32% of respondents would change their production orientation if meat consumption were lower
Other food system actors’ perspectives	Acceptance and willingness to regionalize Need for people to follow seasonality in their diet, especially in the winter Labelling needs more transparency and be included in the gastronomy sector	High acceptance of organic food High price of organic food as an impediment for certain groups of people and gastronomes Need for incentives for farmers and other actors	Meat reduction would allow people to purchase more organic and regional food, if cooking from scratch Meat reduction seems to be the most difficult to implement and to raise awareness about

The bio-physical modelling of Vienna’s urban food system showed that in 2015 this city was drawing on 639,000 ha of agricultural land to provide food to its 1.8 million inhabitants, which represents an area fifteen times the city itself. We estimated that only 8% of Vienna’s land footprint^9^ was located in the regional hinterland and 24% in the rest of Austria. Hence, two-thirds of the footprint covered foreign land with 49% in the European Union and 18% in the rest of the world. We found that the production of food consumed in Vienna caused GHG emissions of 2.29 Mt CO_2_e/year over the whole supply chain. Thereof, agricultural activities (soil management, enteric fermentation of ruminants, emissions from manure and fertilizer application) emitted 60%, food processing 20%, transport of food 12%, emissions related to upstream processes of the production of agricultural inputs 6% and fisheries and aquaculture contributed 2%.

The effect of a regionalization of Vienna’s urban food system, i.e. a change to products from agriculture in the immediate hinterland as far as possible, reduced, first, the land footprint by 21% due to the higher efficiency of Austrian agriculture compared to the other import countries, and, second, the transport demand and hence the transport emissions of food products by one-half. However, due to the small contribution of transport emissions to the total GHG footprint, the effect of a regionalization on the overall GHG footprint was moderate (by 12%, Table 4). Vienna’s urban food system actors showed a generally high acceptance of regionalizing food supply and also mentioned the relevance of including other food systems activities beyond food production (e.g. processing). Not surprisingly, this is the most attractive dimension of food systems change for farmers in the region and is also best accepted by the Viennese population (Table 4).

Farmers can best cope with an increase in the demand for regional products as farm adaptations, in this case, would be incremental rather than transformative. In other words, farmers do not need to change their farm type or operational main focus but the quantity of production. From a consumption perspective, the majority of respondents consider the consumption of regional food as beneficial for the environment (80%), while between 40 and 50% seem not to be aware of the implications of meat and organic food production for the environment. This contrasts with results from the bio-physical analysis which shows that dietary changes towards a reduction of animal-based products have the largest impact on reducing the urban footprint—i.e. between 21 and 35% less land would be needed and it would reduce GHG emissions by 9–33% (Table 4). Such evidence signals the importance of education and of informing people about the impacts of their diets, which was also mentioned by interviewees.

Education programs, while perceived as relevant for promoting sustainable diets, were not considered sufficiently by interviewees, who pointed to the higher costs of sustainable diets as the main barrier to their implementation. Particularly in the case of organic products, we found that farmers are confronted with a higher workload, while additional subsidies and higher producer prices also increase the total gross margin. Whether organic farm conversion is acceptable to farmers—considering higher gross margins but also higher workload—must be evaluated on a farm-by-farm basis, as farmers may face difficulties in organizing the workload due to limited labour available on-farm and lack of skills. In general, half of the farmers indicate the intention to change to organic production, if demand increases. According to farmers, barriers to adapting towards organic farming include particularly the success with the current farm type and the intention to maintain the current income. For the Viennese population, the potentially higher prices of organic (and regional) food do not seem to have a strong influence on respondents’ willingness to increase consumption of these products. However, the price may be a barrier when translating positive attitudes into behavioural intention, as indicated in the comments sections of the consumption surveys. Higher costs of sustainable diets were also mentioned by interviewees as the main reason for low interest in sustainable diets in the private sector. In contrast, interviewees involved in public food procurement consider that it is possible to implement sustainable diets under tight budgets if planned appropriately—i.e. cooking from scratch, reducing meat portions, or avoiding food waste—as several public canteens in Vienna and other cities have proved possible (Morgan and Sonnino 2008; López Cifuentes et al. 2021). The bio-physical modelling revealed that a shift to organic products without changing the dietary composition would reduce the GHG footprint by 18% but increase land requirements considerably (by 57%) due to lower yields and more extensive livestock farming in organic farming systems (Table 4).

Finally, a reduction in meat consumption seems to be the least popular among all actors of Vienna’s urban food system. First, people are less willing to reduce meat consumption than to increase regional or organic food. While half of the respondents agree on the relevance of reducing meat consumption for the environment there is also a considerable share (16.6%) who completely disagree. Cultural values, gender and—to a lower extent—the perceived lower price of meat compared to its alternatives are factors that affect people’s attitudes towards a reduction in meat consumption (Table 4). Second, based on their preferences, livestock farmers see decreased meat consumption as an impediment to the continuation of their businesses. This shows that livestock farmers would usually be more adversely affected by the change in demand and, consequently, more likely to reduce their supply or adapt their production orientation. According to farmers’ intended adaptations, meat production in the region would decline by 26% if meat demand declines. Finally, most interviewees agree on the relevance of reducing meat consumption for the environment and people’s health; yet, actors in the private sector are reluctant to promote and implement this dimension due to the fear that people may not accept it, or it may translate into higher expenses, especially in the gastronomy sector.

The bio-physical modelling, however, shows that the reduction of meat and dairy products has a much larger potential to reduce the GHG and land footprint of the urban food system than the more favoured regionalization or a shift to organic products. Our calculations show that a planetary health diet with both reduced meat and dairy products, for example, can reduce the land and GHG footprint by roughly one-third compared to the current values (Table 4). It also indicates that combining the three dimensions of sustainable diets would significantly reduce GHG emissions of the urban food system and keep land requirements stable while still allowing for a certain share of animal products in the diet. In addition, this would allow profiting from the broad ecological benefits of organic farming. According to the interviews, the city administration is already making steps towards the implementation of such a diet in Vienna by, for example, implementing sustainable public food procurement programs, supporting the food policy council, creating education and awareness campaigns, or further developing farmers markets. Even if the city’s efforts are limited by the European and national legal frameworks as well as the perceived public opinion (especially concerning meat consumption), they seem to promote and support the development of more sustainable local food systems (López Cifuentes et al. 2021).

## Inter- and transdisciplinary knowledge integrated via scenarios

Using the different types of knowledge generated in the project (i.e. system analysis, modelling, surveys), we developed three scenarios comprising different dimensions of sustainable diets that were not assessed further in quantitative terms. Each scenario started with a certain share of meat and milk products in the diet as used in the bio-physical model. The diet in the ‘Ecological Scenario’ is characterized by reduced levels of meat (− 79%) and dairy (− 23%), whereas in the ‘Localized Food Democracy Scenario’, reduced meat consumption (− 66%) is compensated by increased dairy intake (+ 68%), all compared to the current Austrian diet (‘Diet as Usual Through Technology Scenario’) (Table 5). Each scenario generated a narrative that takes a reasonably positive look at the future as the scenarios are supposed to support a transformation towards a sustainable urban food system.

**Table 5:** Overview of the three developed scenarios and exemplary measures for transition pathways

	Ecological Scenario	Regionalized Food Democracy Scenario	Diet as Usual Through Technology Scenario
Diet orientation	Meat: 15 kg/year; dairy: 88 kg/year; no orientation to regionality; maximum organic share	Meat: 22 kg/year; dairy: 195 kg/year; maximum regionality; organic increases depending on commodity groups	Meat: 65 kg/year; dairy: 110 kg/year; if regional, mainly from Austria, not from geographical proximity; organic share remains the same
Narrative	Focus on organic food. Need for more international food imports and efficient production structures outside the city. Higher consumption of legumes. Transparency in the catering industry increases. Institutional, legal and economic support for digital-based social innovation enables farmers to switch to organic	Focus on regionalization with strong involvement of civil society. The demand for protein is compensated by the subsidized production of milk and legumes. Local policy promotes local farms, businesses and alternative networks. Civil society initiatives facilitate access to regional food	Via technological innovation on meat substitutes, diet stays the same. Ethical considerations of animal welfare also resonate. Local politicians and food retailers focus on transparency. The food service industry enable absolute traceability via digital tools. In public food procurement, meals remain inexpensive
Sector	Transition pathways—exemplary measures		
Agriculture and processing	Product-related premium for farmers during organic conversion; taxes for greenhouse gas-intensive products/processes	Warehouse logistics (e.g. food hubs) for small businesses; tax relief and more attractive working conditions for small businesses	Sustainable intensification of agriculture (arable farming, livestock farming) through green subsidies; product-related promotion of GMOs
Distribution and retail	Promotion of climate-neutral means of transport; expansion and promotion of Viennese organic markets through city policy	Tax and legal regulations for alternative forms of distribution; diversification of companies/forms of organization in distribution/trade	Block chain, brands and seals of approval create transparency and trust; awareness raising for alternative technologies and vegan alternatives; promotion of digital retail platforms
Gastronomy and communal catering	Education regarding sustainable cooking, purchase and avoiding food waste; promotion of sustainable cooking skills	Creation of logistics structures (e.g. food hubs) for regional food; food labelling for regional and seasonal food	Block chain provides information on origin and technology; promotion of traditional Austrian cuisine
Consumption	Making organic food available to low-income groups (e.g. vouchers); knowledge building on health/sustainability diets	Promotion of direct relations builds trust and transparency; promotion of cooking skills of regional/seasonal food (e.g. in schools)	Brands and seals of approval create transparency and trust; promotion of acceptance of new food technologies through social events
Cross-sectoral	New rules allow administrative flexibility for experimentation spaces; upscaling of promising initiatives for food saving	Tax incentives for startups/community benefit businesses through local policies; promotion of self-organized initiatives	New technologies allows administrative flexibility for experimentation spaces; tax incentives for technological innovations in circular economy

The ‘Ecological Scenario’ gives priority to organic products and promotes transparency in food systems and the education of and information for the Viennese population. Regional food is not the focus of this scenario and decision-making is based on a top-down hierarchy. The general conversion to organic farming as sketched in the ‘Ecological Scenario’ would result in lower yields per ha and requires more agricultural land. Therefore, this scenario highlights the relevance of decreasing meat and dairy consumption to keep food self-sufficiency rates and avoid externalizing negative environmental effects to other parts of the world via imports. The scenario also assumes additional government support to make the scenario feasible for producers (e.g. new technologies, economic-ecological efficiency) and affordable for vulnerable groups (Table 5).

In contrast, in the ‘Regionalized Food Democracy Scenario’, civil society is assumed to play a crucial role in decision-making and supporting disadvantaged groups. The shift to regional food is associated with higher prices. This is only plausible with great commitment, consequent labelling of food origin, solidarization of civil society and improved food literacy—the latter also concerning a transition from animal to plant-based protein. This scenario also needs local and regional administrative and physical structures that facilitate regional farmers’ and businesses’ activities—e.g. supportive legal and tax regulations, food hubs and other infrastructures supporting the localized food system (Table 5).

Finally, the ‘Diet as Usual Through Technology Scenario’ focuses on technological solutions improving the ecological and economic performance of food systems and meat substitutes allowing for keeping the current Austrian diet. The stakeholders question the robustness of this scenario because of the significant dependence on a small number of technologies such as blockchain or laboratory-grown meat (several of them not yet fully developed and understood regarding their consequences) and a limited number of powerful industry players. Yet, some technological measures were identified that could also support the other two scenarios, e.g. digitalization or creating trust through technological systems that improve transparency in food systems or favouring the development of technologies in circular economies (Table 5).

## Discussion

### Critical reflection on urban scope of action and transition pathways towards more sustainable diets

This paper shows that scenarios are a useful planning tool for exploring urban scopes of action in the context of agricultural and food issues and possible futures and transition pathways. Across different sectors, involving different disciplines and actors, heterogeneous knowledge, qualitative and quantitative data, but also very different ideas about the future can be brought together (Chermack and Lynham 2002; Wiek et al. 2006). These scenarios served to integrate empirical scientific research on the status quo of Vienna’s urban food system and to identify possible transition pathways towards a more sustainable diet. The developed scenarios take a reasonably positive look at the future, show the complexity of urban food systems transformation towards sustainability and highlight the different trade-offs. Thus, several futures can be juxtaposed and discussed comparatively.

According to the ‘Ecological Scenario’ and the ‘Diet as Usual Through Technology Scenario’, local food production could only play a minor role in the future of Vienna. As argued by Deelstra and Girardet (2000), measures to secure the food supply would probably be needed to ensure a supply even if international supply chains fail due to protectionist measures by food-exporting countries, armed conflicts or a logistics crisis. Such measures could include diversification of supply regions, storage, the release of public green areas for food cultivation, or long-term regional supply contracts. The ‘Regionalized Food Democracy Scenario’ assumes a possible expansion of food production and processing activities in Vienna. In terms of planning, the challenge here is to expand priority areas for agricultural production and to gain additional areas for micro-gardening through roof and courtyard greening, or the deconstruction of brownfield sites, but also to secure areas for food processing and markets. As argued by McClintock et al. (2013), this requires identifying and negotiating the varied interests of multiple stakeholders. In addition, more attractive framework conditions would be needed for all those who might be interested in a profession in the food supply sector in the future or social measures to support disadvantaged groups in the face of rising food prices. In line with Feola (2020, p. 5), this scenario shows that “peri-urban spaces are economically multifunctional, socially diverse and ecologically complex” and thus “no one-size-fits-all policy is effective for governing [urban food and agriculture].”

This complexity is also reflected in the knowledge co-production of this project. For example, by combining different disciplines and actors’ perspectives, our transdisciplinary multi-method approach has revealed that the popularity and the positive environmental effects of dietary changes are diametrically opposed. While the bio-physical model showed that a reduction in meat consumption has the largest potential to reduce land and GHG footprints—as previous studies have already shown in other contexts and different scales (see, for example, Godfray et al. 2010; Garnett 2011; Johnston et al. 2014; Seto and Ramankutty 2019)—the Viennese population and other urban food system actors perceived regional food as the most environmentally friendly option. This discrepancy points to the idealization of regional food found in other studies—i.e., the local trap (see, for example, Sonnino 2013; Allen and Prosperi 2016; Moragues-Faus et al. 2017). Interestingly, in contrast to previous studies (see, for example, Barosh et al. 2014), higher prices seem less of an issue for respondents to hypothetical questions than changing dietary patterns: regional and organic foods are related to higher prices at the point of sale and higher income for farmers, but not necessarily with a shift in diets, while reducing meat would mean a major shift in diets. However, this also needs to be considered in light of the hypothetical bias (Cummings and Taylor 1999), which may result in overstating economic values because respondents allocate less importance to budget constraints.

### Critical reflections on knowledge co-production and knowledge gaps

Our study has produced different types of knowledge that have helped us better understand the trade-offs of implementing sustainable diets in an urban food system as shown in the previous section. We used a framework for the ex-post analysis of knowledge co-production (Muhar and Penker 2018), which focuses on the integration of diverse actors’ knowledge and thus on the cognitive dimension—versus the emotional or social-interactional dimensions—of transdisciplinary processes (see Pohl et al. 2021). This framework helped us to reflect on the potential of integrating a manifold of knowledge types but even more on the limitations we encountered along the way.

First, the lack of systematic and accessible data on processing, logistics, retail and gastronomy concerning Vienna’s urban food system could only partly be mitigated via interviews and expertise in the advisory board by representatives from these sectors. Furthermore, our surveys focused on farmers and the general population, omitting the processing, logistics, retail and gastronomy actors in between. We do not know if additional surveys would have helped to close the data gap, given the power realities within food systems, which also define who is obliged/willing to provide data and who is not (e.g. retailers not sharing their data with statistical offices). Nevertheless, the project results are limited due to these knowledge gaps, which calls for more systematic monitoring of urban food supply and future research on these sectors.

Second, counterfactual thinking proved difficult, as anything/anyone is connected to almost everything/everyone in polycentric food systems (Johnston et al. 2014; van Bers et al. 2019; Marshall et al. 2021). The complexity of these systems that are governed by diverse interrelated decision-making centres at multiple levels, by globally interacting businesses and diverse governments with overlapping jurisdictions and civil society groups with conflicting goals for animal welfare, climate, biodiversity, health, culinary heritage and social equity, challenges analytical approaches that try to selectively focus on specific dietary changes.

Third, the integration of these different types of knowledge enriched the results of the project: (1) the stakeholders’ experiential knowledge helped to fill sectoral data gaps but also data gaps on plausible futures that might be rather shaped by the stakeholders’ expectations of their own future food production and consumption practices than by past patterns depicted in empirical data; (2) their context-specific knowledge supported the identification and selection of innovative initiatives with transformative potential, but also barriers of change in Vienna; (3) the stakeholders provided context-sensitive assessments of the consistency and robustness of the scenarios; and (4) strategic knowledge on whom to involve at what stage of the project. In the opposite direction, the stakeholder dialogue was enriched with empirical evidence that highlighted the bio-physical and agricultural-economic boundaries and trade-offs to be acknowledged when discussing urban food system change. The surveys and interviews provided a broader perspective on the scope of changes accepted by the local population and manageable by local farms and on the opportunities and barriers of scaling food sustainability initiatives. In line with previous research (Pohl et al. 2010; Raymond et al. 2010; Enengel et al. 2012), we assume that the co-production process has resulted in knowledge that is more robust, broadly legitimised and better tailored to the local context. Yet this co-production process was challenging and limited at times. One of the key challenges was the transdisciplinary integration of modelling key variables based on historic data and ceteris paribus assumptions with a broad and future-oriented perspective of the participatory process. The communication of scientific knowledge (e.g. with all its limitations, underlying assumptions, uncertainties, etc.) to other actors so that it was accessible and understandable was also problematic at times and probably limited.

Fourth, although the inclusion of diverse types of knowledge from various actors added a more integrative perspective on Vienna’s urban food system’s complexities, their knowledge contribution varied for the different project phases. While the core scientists and consultants mainly contributed and developed scientific knowledge, other actors provided experiential, context-specific, phenomenological, and strategic as well as scientific knowledge. Stronger and broader involvement of strategic case actors already in the project development and problem framing (Lang et al. 2012) might have resulted in project outcomes better tailored to the actors’ needs and might have created more backup for a bigger participation process. Unfortunately, as is often the case, there was only limited time and a lack of financing for the project development which limited the involvement of different actors at this early stage. We tried to partly compensate for this limitation by providing a space for the discussion of the project design and goals in the first meeting in May 2018.

For critical project decisions such as defining Vienna’s urban food system, interviewee and case selection, questionnaire development, or the interpretation of results, the advisory board and further actors of the Viennese food policy council willingly contributed their rich expertise. The project involved a diversity of actors from outside of academia covering the different sectors of food systems—i.e. production, retail, distribution and civil society—yet some perspectives were still missing in the process. For example, disadvantaged groups were only integrated very late to include their perspectives on sustainable diets and in the advisory board, there were no climate change deniers or heavy meat eaters. These were possibly integrated as data providers in surveys, but not in consultative or co-creation tasks. This allowed for a protected space for developing future perspectives and ideas, however, the limited size and heterogeneity of the advisory board might have decreased the impact of the joint learning process—i.e. there was more confirmation of previous knowledge and only a few surprising learnings for individual scientists and actors involved, such as the local scope of action being bigger as initially expected.

Finally, the formation of the Viennese food policy council and their work on a food strategy with the City of Vienna briefly opened a window of opportunity for linking the scenario process planned for transdisciplinary knowledge integration with a broader participatory process involving more heterogeneous actors. However, the city administration finally opted against a broader participation process for a process that would have included businesses and city departments far beyond the transdisciplinary team of researchers and advisory board due to organizational reasons, political pressure, COVID-19, the changed situation due to local elections, or a combination of all. Thus, we organized a down-sized process combining information, consultation and co-decision-making among the narrow circle of the advisory board and researchers. While representatives of the city administration department responsible for coordinating food policies in the City of Vienna were involved, other local government departments (responsible for health institutions, schools, agriculture, land-use planning, gastronomy and tourism) were not included.

This transdisciplinary approach to the scenarios and the food strategy probably is too thin and might not have the broad ownership and support needed for leveraging a food systems transformation as aimed by the food strategy and the Milan Urban Food Policy Pact. However, the results and scenarios developed, the exchange across disciplinary and sectoral boundaries and the measures co-developed in workshop 11/2020 have informed the first Vienna Food Strategy, which representatives of the City of Vienna and the Viennese food policy council are currently discussing with numerous stakeholders. In addition to a common vision and very concrete and time-bound measures, this strategy also contains indicators for monitoring the achievement of objectives. To what extent this food strategy can actually activate urban actors for the realization of the vision of sustainable diets in Vienna remains to be seen.

## Conclusions

This paper shows the diversity of actors and their different types of knowledge needed for exploring urban scopes of action and transition pathways in the context of agricultural and food issues. It discusses the challenges that come with the integration of these different actors’ knowledge, methods and epistemologies.

This inter- and transdisciplinary approach helped us to untangle the complex issue of sustainable diets. This study has shown that while empirical data highlights the reduction of meat consumption as the largest potential to reduce the environmental effects of Viennese diets, Vienna’s population is reluctant towards changing their diets and perceives regional food as the most environmentally friendly option. The three scenarios address and partly resolve the trade-offs of implementing sustainable diets. They illustrate the pros and cons of diverging futures and the interactions between single dimensions of sustainable diets. These scenarios show plausible, but extreme futures. However, it will most likely not be either one or the other. Many components of these scenarios could complement each other and several different food systems are probably needed to cope with the heterogeneity of production and consumption preferences and possibilities. Although the scope for action at the local level was perceived as limited by actors, the discussion on the scenarios showed that it is greater than originally thought. Local actors do not have to wait for changed European Union or national framework conditions but can transform urban food systems through public procurement, labelling, education, supporting innovation, influencing meta-discourses and networking also at the local level. This, however, requires coordination across diverse groups, interests and types of knowledge to overcome lock-ins.

## Data Availability

The data that support the findings of this study are available from the corresponding author upon reasonable request. However, due to the sensitive nature of the research, supporting data may be only partially available.

## Footnotes

1 https://sdgs.un.org/2030agenda.

2 http://urbanfood.boku.ac.at/.

3 https://www.milanurbanfoodpolicypact.org/.

4 https://www.organic-cities.eu/.

5 A food policy council is a civil society association with members from various parts of an urban food system from production up to consumption that aims to make food systems more sustainable and democratic (https://ernaehrungsrat-wien.at/).

6 The multi-level perspective differentiates three levels to understand the complex interacting developments in food systems (see Geels 2002).

7 Caritas is a large aid organization in Austria and Vienna, which is engaged in various projects with vulnerable or marginalized groups: https://www.caritas-wien.at.

8 A preliminary analysis was performed to ensure that the regression model does not violate the assumptions of linearity, multicollinearity and homoscedasticity (Field 2009). The MLR models are a statistically significant fit of the data, as indicated by F test statistics of 4.447 (SR), 8.022 (SO) and 8.320 (SM) and a *p* value less than 0.05. The models account for 13.8% of the variance in the intention of consuming mainly regional food, 19.2% of the variance in the intention of consuming mainly organic food and 17.1% of the variance in the intention of consuming a maximum of 400 g of meat per week.

9 We define the land footprint as the sum of land area (cropland and grassland) required for the production of food consumed in Vienna. The GHG footprint comprises all emissions linked to the different production, processing and transport steps associated with urban food consumption.
